# Neural Substrates of External and Internal Visual Sensations Induced by Human Intracranial Electrical Stimulation

**DOI:** 10.3389/fnins.2022.918767

**Published:** 2022-07-22

**Authors:** Yanyan Li, Zheng Tan, Jing Wang, Mengyang Wang, Liang Wang

**Affiliations:** ^1^CAS Key Laboratory of Mental Health, Institute of Psychology, Beijing, China; ^2^Department of Psychology, University of Chinese Academy of Sciences, Beijing, China; ^3^Sanbo Brain Hospital, Capital Medical University, Beijing, China

**Keywords:** offline perception, intracranial electrical stimulation, epilepsy, hippocampus, inferior temporal cortex

## Abstract

Offline perceptions are self-generated sensations that do not involve physical stimulus. These perceptions can be induced by external hallucinated objects or internal imagined objects. However, how the brain dissociates these visual sensations remains unclear. We aimed to map the brain areas involved in internal and external visual sensations induced by intracranial electrical stimulation and further investigate their neural differences. In this study, we collected subjective reports of internal and external visual sensations elicited by electrical stimulation in 40 drug-refractory epilepsy during presurgical evaluation. The response rate was calculated and compared to quantify the dissociated distribution of visual responses. We found that internal and external visual sensations could be elicited when different brain areas were stimulated, although there were more overlapping brain areas. Specifically, stimulation of the hippocampus and inferior temporal cortex primarily induces internal visual sensations. In contrast, stimulation of the occipital visual cortex mainly triggers external visual sensations. Furthermore, compared to that of the dorsal visual areas, the ventral visual areas show more overlap between the two visual sensations. Our findings show that internal and external visual sensations may rely on distinct neural representations of the visual pathway. This study indicated that implantation of electrodes in ventral visual areas should be considered during the evaluation of visual sensation aura epileptic seizures.

## Introduction

Offline perception (Fazekas et al., 2021), such as those occurring during a hallucination, imagery, mind-wandering, and dreaming, is self-generated sensations without physical stimuli, yet their neural correlates are deceptively similar to those of stimulus-driven perception (Mason et al., 2007; Horikawa et al., 2013; Pearson and Westbrook, 2015; Pearson et al., 2016). Offline perception can be, but does not have to be, accompanied by the feeling of presence (Fazekas et al., 2021). For example, individuals can be fooled into thinking that the content of the offline perception is indeed in front of them, such as hallucination; in other cases, individuals cannot be normally fooled into feelings of presence, such as imagery. This raises the essential question of how the brain identifies whether the stimuli of the senses exist in the external world. It is critical to explore the nature of conscious experiences and their important clinical implications for psychopathology. Offline perception can involve any of the senses (Dijkstra et al., 2019; Pearson, 2019), but here we focus on visual sensations given that we, as humans, are intensely visual creatures.

One method that may provide useful insights into this issue is intracranial electrical stimulation (iES) in awake neurosurgical patients, such as patients with drug-resistant epilepsy and implanted electrodes for seizure zone evaluation. IES probably provides the best way of causally perturbing brain function in humans, and it has long been known to induce various yet replicable perceptual and behavioral phenomena (Selimbeyoglu and Parvizi, 2010). For example, stimulations of the occipital, occipitotemporal, occipitoparietal, inferior, and middle temporal areas induce both the simple visual sensations, such as seeing simple patterns, spots, shapes, a blob of flashing, light, colors, movement, or phosphenes in the external world (Murphey et al., 2009; Jonas et al., 2014a,b, 2018; Rangarajan et al., 2014; Winawer and Parvizi, 2016; Bosking et al., 2017), and complex visual sensations such as seeing people or scenes (Megevand et al., 2014; Andelman-Gur et al., 2020). In contrast, stimulations of the medial temporal lobe and middle or inferior frontal gyrus are associated with a visual dreamy state (Blanke et al., 2000; Vignal et al., 2007) and novel not-previously experienced imagery (Lai et al., 2020).

Based on previous studies, we hypothesize that offline perception may engage different neural networks when it is accompanied by the feeling of presence or absence. Specifically, external visual sensations involve a neural network within the visual cortex, whereas internal visual sensations may engage the medial temporal lobe. However, to the best of our knowledge, no study has directly explored this issue by comparing neural substrates between visual sensations accompanied by the feeling of presence and those that are not.

Notably, a dreamy state and déjà vu are described primarily as vivid mnemonic experiences. They are not only visually imaged, but also (re)lived by the participants, sometimes with emotional content. Briefly, a dreamy state and déjà vu are richer experiences than that pure visual sensations. Hence, the internal visual sensations in the current study encompassed only imagery. Here, we investigated the neural substrates of external and internal visual sensations by analyzing the effects of high-frequency electrical stimulation and further compared the neural differences between them.

## Materials and Methods

### Participants

The inclusion criterion (CRI-1) to enter the study was that at least one type of visual response (either external or internal visual sensations) was elicited by electrical stimulation. Forty patients (mean age 24.98 years; 15 females), out of 144 patients (mean age 21.63 years; 54 females) with focal drug-resistant epilepsy, met these criteria and underwent video stereoelectroencephalography evaluation to define the cerebral structures involved in the onset and propagation of seizure activity from January 2017 to December 2019 at the Sanbo Brain Hospital, Capital Medical University, China.

Additionally, we adopted a stricter inclusion criterion (CRI-2) as a control to validate the main results. To account for the potential influence of epilepsy directly or indirectly on the visual response to electric stimulation, patients were excluded: (1) if they were in their second operation or reported hallucination aura before the epileptic seizure and (2) if the implanted electrodes were in the epileptogenic zone identified by the neurologists (JW, MYW).

All the patients, or their guardians, provided informed consent for the surgical procedure and the review of data for scientific purposes. This study was approved by the Ethical Committee of the Institute of Psychology, Chinese Academy of Sciences (ID H20034).

### Intracranial Electrical Stimulation Procedure

Intracranial electrical stimulation was used to elicit some or all of the electroclinical seizures and map the cortical functional areas. IES was performed by delivering biphasic electrical stimuli (Nicolet Cortical Stimulator, Wisconsin, United States) with a pulse width of 0.3 ms and a duration of 5 s. IES frequency was 50 Hz (high-frequency iES). Stimulation was stopped at the onset of clinical response (e.g., preseizure aura and epileptic seizure) or the appearance of electroencephalography (EEG) after discharges. The stimulation intensity ranged from 0.5 to 6 mA. For each stereo-EEG electrode, contiguous couples of contacts were tested according to a clinical inquiry using a bipolar montage. The intensity of stimulation was raised in 0.5 mA steps. The patients were “blind” to the timing and stimulated location.

**FIGURE 1:**
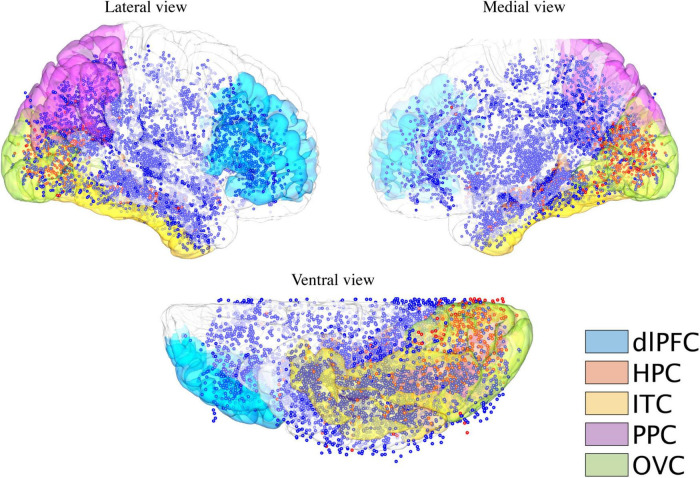
Cortical distribution of electrodes receiving intracranial electric stimulation. Electrodes were stimulated during the functional mapping sessions over all the patients (*n* = 40 patients) pooled on the MNI152 brain surface. Blue dots indicate stimulated electrodes, which elicit no visual responses, while red dots indicate stimulated electrodes, which elicit either internal or external visual responses for at least one patient. The structural modules are rendered on the brain’s surface with different colors. dlPFC, dorsal lateral prefrontal cortex; HPC, hippocampus; ITC, inferior temporal cortex; PPC, posterior parietal cortex; and OVC, occipital visual cortex.

After excluding electrodes without electric stimulation under CRI-1, 110 ± 17.4 electrodes remained for each patient, with a total of 6,272 stimulated contacts for the 40 patients (Figure 1). Alternatively, 32 patients and approximately 102.4 ± 14.8 electrodes per patient (5,020 contacts in total) remained under CRI-2 for each patient.

### Operational Definition of External and Internal Visual Sensations

We classified external and internal visual sensations based on the subjective reports of the patients. If the patient reported imagery of something/somebody or clearly informed seeing something/somebody in his/her mind, we categorized them as internal visual sensations. For example, we classified responses such as “I saw something flashed in my mind” (Supplementary Video 1) as internal visual imagery. Notably, we did not include reports of vivid autobiographic memories or déjà vu. In contrast, if patients reported seeing images using their eyes or in front of them without physical stimuli (not a deformation of a real object that was excluded in this study), we classified these responses as external visual sensations. Furthermore, patients reported seeing the images, as if they were in front of them. Simultaneously, patients also realized that nothing was actually presented; for example, “I saw something like a grain of water in my right eye” (Supplementary Video 2). In the current retrospective study, despite the absence of structured interviews, the clinician confirmed the contents of the oral report with the patients during the iES.

In addition, we divided the external visual sensations into simple or complex sensations based on the nature of the reported visual stimuli (Selimbeyoglu and Parvizi, 2010). If the patients reported that they perceived objects, animals, persons, or scenes, we assigned these reports to complex external visual sensations. In contrast, if the patients reported seeing something such as phosphenes, shapes, colors, sizes, depths, flickers, and motions, we assigned them to simple external visual sensations.

### Stereotactic Implantation and Contact Localization

All the patients were chronically implanted with depth electrodes (0.8 mm in diameter; Beijing Huakehengsheng Healthcare Corporation Ltd., Beijing, China), featuring 8 to 16 contacts, 2 mm long, and 1.5 mm apart. The number of electrodes and sites of implantation were established based on the clinical electroencephalographic features of the seizures recorded during long-term video-EEG monitoring. To reach the clinically relevant targets, the stereotactic coordinates of each electrode were calculated preoperatively using individual MRI.

Electrodes’ coordinates in the individual volume space and the Montreal Neurological Institute (MNI) 152 template spaces were obtained using a semi-automated intracranial electrode localization toolbox (Qin et al., 2017). Briefly, the patient’s preoperative MRI was co-registered with the postoperative CT. After the CT image was aligned to the MRI, the electrode clusters were automatically detected and manually labeled according to the clinically designed trajectories. Then, the spatial coordinates of each contact were generated in the individual volume space. To visualize the exact locations of the electrode contacts with higher accuracy, we reconstructed the electrode trajectories on the individual brain surface. To register the coordinates of all the electrodes into a standard space, the patient’s MRI was normalized to the standard MNI template using a combined volumetric- and surface-based registration algorithm (Postelnicu et al., 2009). The transformation was then applied to all the contacts to generate electrodes’ coordinates in the MNI standard space. Coordinates for the electrodes located in the left hemisphere were mirrored in the right hemisphere for a simplified display.

### Structural Modules

To explore the spatial distribution of brain responses (external and internal visual sensations) to electrical stimulation and draw a general conclusion at the level of brain regions, this study focused on several structural modules according to previous studies, each of which comprised several structural regions identified by FreeSurfer software^1^ : occipital visual cortex (lateral occipital cortex, lingual, cuneus, and pericalcarine sulcus), dorsal lateral prefrontal cortex (inferior frontal gyrus and middle frontal gyrus), inferior temporal cortex (fusiform area and inferior temporal gyrus), posterior parietal cortex (inferior parietal lobe and superior parietal lobe), and hippocampus (Figure 1). To determine which module an electrode contact belonged to, a sphere with a radius of 8 mm was generated at the center of the contact coordinate. The module-based distribution of electrodes that were electrically stimulated and induced by either type of visual response is shown in Table 1. Electrodes that were not located in any of these modules were assigned to “others.”

**TABLE 1:** Elicitation rates and current thresholds.

Regions	Electrode contacts		Stimulation current thresholds (mA)^a^	
	Total	Visual response	Mean minimum stimulation current threshold mA (s.d.)	Mean maximum stimulation current threshold mA (s.d.)
Occipital visual cortex	506	252 (49.80%)	2.64 (2.05)	3.35 (2.18)
Inferior temporal cortex	446	38 (8.52%)	3.99 (2.02)	4.61 (1.81)
Posterior parietal cortex	542	73 (13.47%)	3.65 (2.14)	4.31 (1.95)
Hippocampus	438	46 (10.50%)	2.87 (1.83)	3.75 (1.94)
Dorsal lateral prefrontal cortex	434	0 (0%)	4.52 (1.95)	5.21 (1.52)
Others	3,906	149 (2.37%)	3.58 (2.13)	4.25 (2.00)
Totals and means	6,272	558 (8.91%)	3.55 (2.13)	4.24 (2.00)

*^a^Note that the current threshold here is the threshold of current intensity used during iES, including both responsive and unresponsive contacts.*

### Response Rate to Electrical Stimulation

First, the response rate was calculated for separate visual response types and pooled together on the cortical surfaces and in different brain modules. For cortical surface-based calculations, the cortical response rate at each vertex surrounding the contact center with an 8 mm radius sphere was calculated as the number of stimulated electrodes eliciting visual responses divided by the number of all the stimulating electrodes. For module-based calculations, a module’s response rate was calculated as described above, but was limited within each module.

### Predominant Response in the Brain Modules

A binomial test was used to perform a statistical test to reveal the predominant module-specific visual response to electrical stimulation. The ratio of the number of electrodes inducing one specific response to the total number of response-inducing electrodes [i.e., external visual sensations/(external visual sensations + internal visual sensations)] within each module was compared to the expected value calculated based on the whole cerebral cortex.

### Overlapped Hierarchical Processing for Internal and External Visual Sensations

We further studied whether the probability of detectable perceptions of a visual area varied with the position of the area in the visual cortical hierarchy for both the internal and external sensations. Hence, the cortical surface was parcellated finely with a recent cytoarchitecture-based atlas (Amunts et al., 2020); then, the label of each contact was determined in the same manner as that described above in the structural module section.

In addition, the Dice coefficient (Dice, 1945; Hoenig et al., 2018) was adopted to calculate the spatial overlap between the two different visual responses within a region of interest with an 8 mm radius to test whether internal and external visual sensations have more overlap in high-level areas. The volume was measured from each of the two binarized images and the region-wise Dice coefficient (D) was calculated as follows:


$$\text{D}=\frac{2\times intersectionvolumesize}{\begin{array}{c} internalsensationvolumesize\\ +externalsensationvolumesize \end{array}}$$

For brain areas that were involved in both the internal and external visual sensations, we reordered D values based on their hierarchical roles in the general visual pathway (Felleman and Van Essen, 1991) to test whether there was a trend consistent with our hypothesis.

### Control Analysis

To ensure that the neural representations of internal and external visual sensations were stable and replicable, we conducted a series of reliability analyses. The specific operations are as follows: (1) validation of the main analysis results for a stricter inclusion criterion (CRI-2); (2) internal and external visual sensations reproducibility for the same eloquent site; (3) whether and how false-positive reports were identified when iES was performed by delivering biphasic electrical stimuli at 1 Hz (low-frequency iES); (4) cross-participant reliability, i.e., whether adjacent parts of the cortex elicited similar types of external visual sensations in different participants and if the cortical distribution of electrodes that elicited internal visual sensations in different participants were similar; and (5) whether complexity could underlie the functional dissociation between internal and external visual sensations.

## Results

### Sex Difference

Among the 40 patients, there were 15 female patients. It is important to consider this sex imbalance in the context of the overall cohort. At least one type of visual response was elicited in 40 (15 females) of 144 (54 females) patients. There were no significant sex differences in the visual responses (chi-square = 0, *p* = 1). External visual sensations were elicited in all the 40 patients, while internal visual sensations were elicited in 7 (2 females) patients. There was no significant sex difference in visual sensation (chi-square = 0.29, *p* = 0.59).

### Probability for Electrical Stimulation to Elicit Visual Responses Varied Across the Cortical Surfaces

**FIGURE 2:**
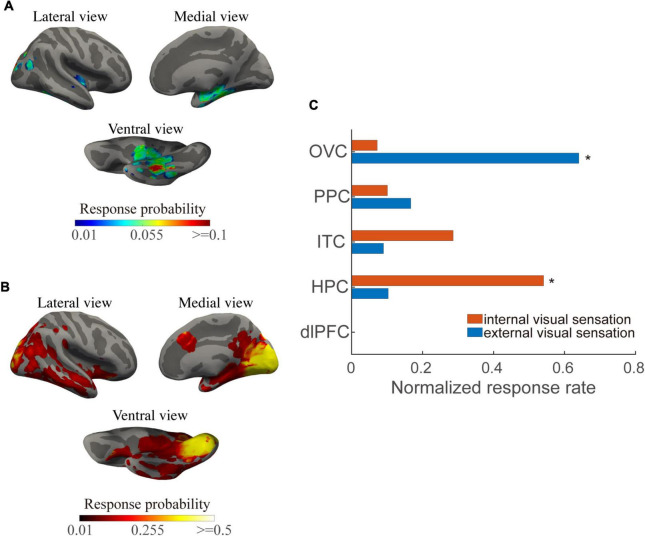
Internal and external visual sensation probability across the cortical surfaces and structural modules. **(A)** Mean response probability for internal visual sensations across the cortical surfaces. **(B)** Mean response probability for external visual sensations across the cortical surface. **(C)** Normalized response percentage, normalized across modules, for each visual response type. Asterisks indicate regions in which the individual chi-square value exceeds a significant level (*p* < 0.01, Bonferroni correction for multiple comparisons).

We first investigated the visual responsiveness across the cortex and modules for the two types of visual responses. The current thresholds that induced internal visual sensations were significantly greater than those that induced external visual sensations (*p* < 0.001). In addition, a significantly unequal probability across cortical modules was found as revealed by the chi-squared test for each visual response. Regions involved in the inferior and medial temporal cortex and posterior cingulate cortex showed enhanced internal visual sensations compared to that of the occipital visual cortex (Figure 2A). Regions with the highest response probability were located in the visual cortex, particularly in the medial part (Figure 2B). These results are compatible with findings that induced external visual sensations varied with the position of the area in the visual cortical hierarchy, with much more effective stimulation of early visual areas than that of stimulation of higher visual areas (Murphey et al., 2009).

At the module levels, normalized responsiveness in the occipital visual area showed a predominance of external visual sensations, while the hippocampus and inferior temporal cortex played predominant roles in the internal visual sensations. Both the types of visual response exhibited moderate responsiveness in the posterior parietal region (Figure 2C). Notably, some areas, such as the posterior inferior temporal gyrus and posterior cingulate cortex, may exhibit visual phenomena either locally or due to current propagation to the occipital cortices.

### Neural Representations Dissociate Internal and External Visual Sensations

**FIGURE 3:**
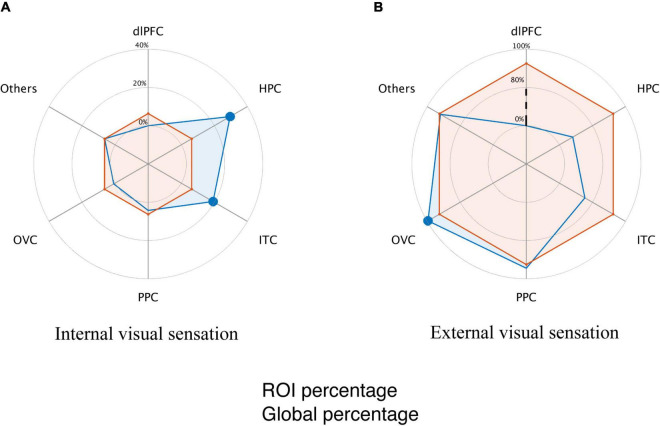
Dissociated brain module involvement in involuntary visual imagery and hallucination. **(A)** Global and module internal visual response percentage. The orange line indicates the average proportion of internal visual responses relative to all the elicited visual responses. The blue line indicates the proportion of internal visual responses within a specific module. **(B)** Global and module external visual response percentage. The orange line indicates the average proportion of external visual responses relative to all the elicited visual responses. The blue line indicates the proportion of external visual response within a specific module. The dashed black line indicates that the response percentage did not scale linearly for illustration. The modules with a filled large circle exhibit a significantly higher percentage than that of the cortical average (*p* < 0.01, Binomial test, Bonferroni correction for multiple comparisons).

Next, we targeted the differences in the brain modules that contribute to internal and external visual sensations. We employed a binomial test with a null distribution, in which the visual response type predominance was evenly distributed across brain modules. Module-specific visual response type was observed. Specifically, external visual sensations dominated the occipital visual area (*p* < 0.001, Figure 3A), while internal visual sensations dominated the hippocampus and inferior temporal cortex (*p* < 0.001 for the hippocampus; *p* = 0.002 for the inferior temporal cortex; Figure 3B). No dominant visual response was detected in the posterior parietal areas.

Given that a large proportion of patients (82.5%, 33 of 40) only reported external visual sensations, it might lead to a biased module-based distribution for visual response-related functional dissociation. Similar findings were reproduced in seven participants who reported both the internal and external visual sensations (*p* < 0.001 for external visual sensation predominance in the occipital visual area, *p* < 0.001 and *p* = 0.003 for internal visual sensation predominance in the hippocampus and inferior temporal cortex, respectively; binomial test), indicating that the dominant visual responses to electrical stimulation were indeed module-dependent.

### Hierarchy of Shared Neural Mechanisms of Visual Imagery and Hallucination

**FIGURE 4:**
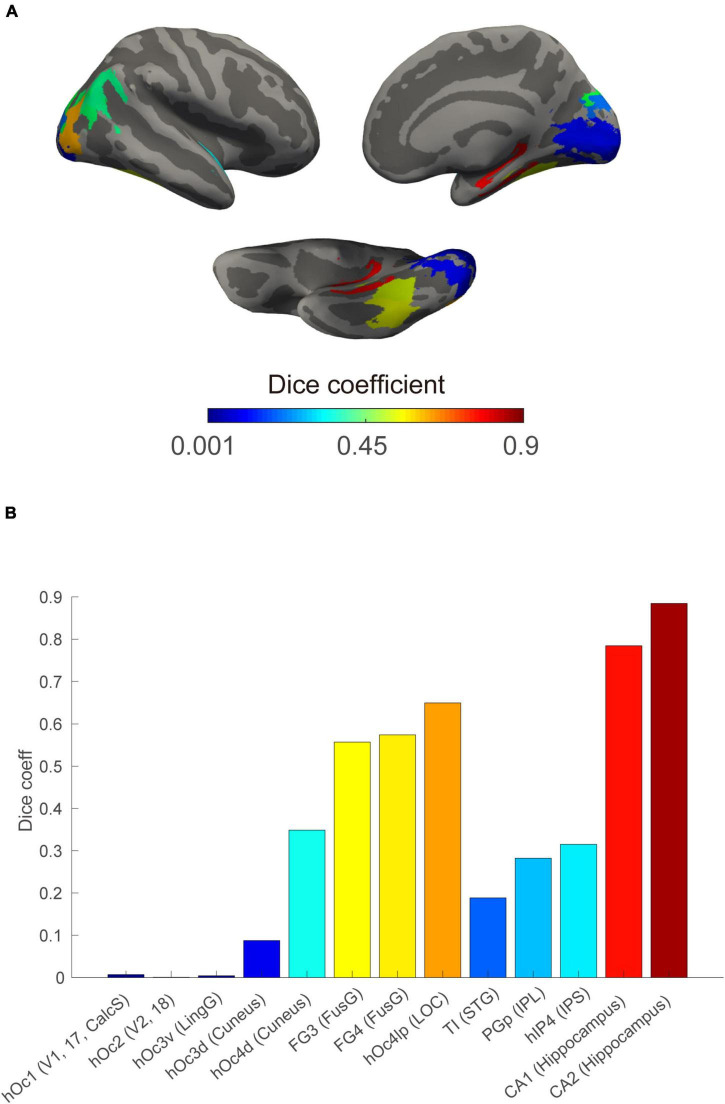
Increased spatial overlap between internal and external visual sensations in regions along the visual processing stream. **(A)** Spatial Dice coefficient between the internal and external visual sensations at the ROI level rendered on the cortical surface based on a public cytoarchitecture atlas. **(B)** ROI-based Dice coefficient along visual information processing stream defined generally. Bars indicate the extent of spatial overlap, displayed using the corresponding ROIs color on the above surface. CalcS, calcarine sulcus; FusG, fusiform gyrus; IPL, inferior parietal lobule; IPS, intra-parietal sulcus; LingG, lingual gyrus; LOC, lateral occipital cortex; and STG, superior temporal gyrus.

Finally, we found an increasing overlap with ascension in the visual processing hierarchy (Figure 4). Internal and external visual sensations have greater overlap in high-level areas (e.g., the hippocampus) than in low-level areas (e.g., V3).

### Reproducibility

**FIGURE 5:**
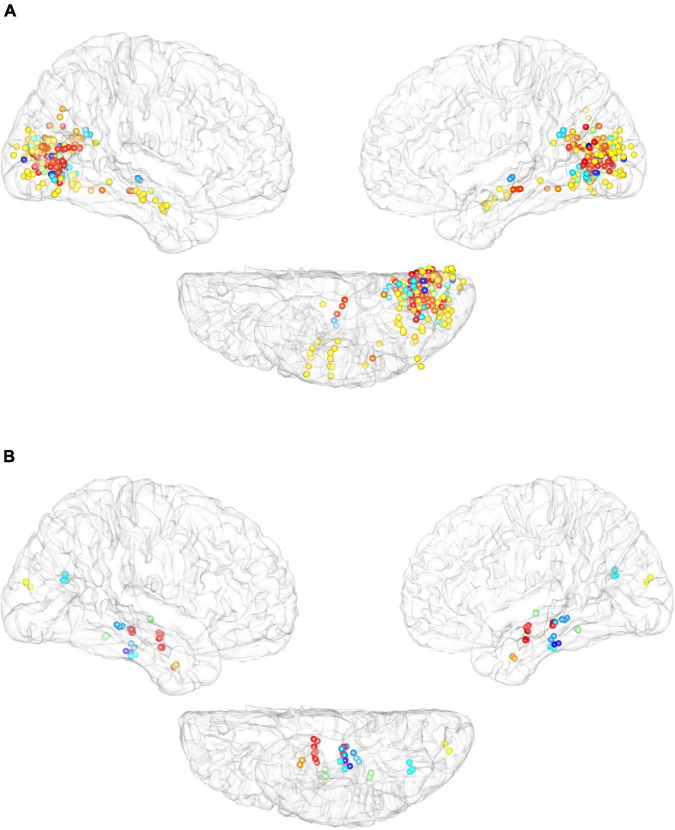
Cross-participant reliability of elicited response to electrical stimulation. **(A)** Cortical distribution of electrodes that elicited external visual sensation of flickers across different participants. **(B)** Cortical distribution of electrodes that elicited internal visual sensations by electrical stimulation. Different color-coded dots encode different participants.

(1) We validated the dissociated brain module (*p* < 0.001 for the visual area, hippocampus, and inferior temporal cortex; binomial test) and increased overlap with ascension in the visual processing hierarchy between the internal and external visual sensations in a stricter inclusion criterion. These findings were reproduced in CRI-2.
(2) We further investigated the reproducibility among participants. Seven patients delivered electrical stimulations by increasing the electrical current density (less than 6 mA) after reporting visual imagery (16 repetitions) and/or hallucinations (145 repetitions). Internal and external visual sensations were repeated at rates as high as 100 and 91.8%, respectively.
(3) To control for demand characteristics and false-positive reports, 1 Hz iES was delivered to eight participants (20%). No reports of internal or external visual sensations were identified even after numerous low-frequency stimulations.
(4) We also investigated reproducibility across participants. Stimulation of adjacent parts of the cortex elicited similar external visual sensations in different participants (Figure 5A, flickers, for example). In addition, we also presented maps of the cortical distribution of electrodes that elicited internal visual sensations in different participants (Figure 5B), which also showed an obvious aggregation effect, which mainly occurs in the hippocampus and inferior temporal cortex.
(5) Finally, we compared neural differences between simple and complex external visual sensations. No significant predominance of simple or complex external visual sensations was found in any of the structural modules, suggesting that distinct neural representations were caused by the inherent differences between internal and external visual sensations, regardless of perceptual complexity.

## Discussion

Understanding how the brain distinguishes an internal stimulus sensation from an external stimulus is critical to understanding the nature of consciousness (Dijkstra et al., 2019). To the best of our knowledge, this is the first study to reveal that stimulating the hippocampus and inferior temporal cortex primarily induces internal visual sensations, while stimulating the occipital visual cortex mainly triggers external visual sensations. Furthermore, we found some overlap between sites that induced internal and external visual sensations emerging as early as the occipital visual cortex and this overlap increased at higher levels of the visual processing hierarchy. This hierarchy of shared neural substrates of internal and external visual sensations fits well with the model of imagery dynamics proposed in a study by Pearson (2019).

In addition, since subjective symptoms were reported by different patients with degrees of differences in age, psychometric, and cultural profiles, the underlying neural mechanisms to dissociate between internal and external visual sensations across different groups might be biased. To minimize this factor, we reanalyzed the data from seven participants who reported both the internal and external visual sensations. The main findings were replicated for this subgroup.

### Visual Cortical Stream

Consistent with our results, the hippocampus has been suggested to support complex or spatially distributed images (Bird et al., 2010). Evidence from single-neuron recordings in humans has revealed hippocampal correlates of internal visual sensations (Kreiman et al., 2000). Internal visual sensations can be triggered by combining different information stored in our memory and it is difficult to imagine something that you have never been exposed to. Thus, the hippocampus has been proposed to be positioned at a location that forms the imagery content (Pearson, 2019). Further studies are needed to elucidate the exact roles of the hippocampus in imagery.

Several internal visual sensation active sites were observed in the occipital and temporal cortices, while external visual sensation responses were found mostly in the occipital cortex. Internal visual sensations seem to involve the corresponding sensory cortex (Pearson and Westbrook, 2015; Pearson, 2019). For example, a previous human imaging study found that color imagery could be decoded in V1 and V4 (Bannert and Bartels, 2013). In contrast, previous anatomical studies have revealed that external visual sensations are related to atrophy in the occipital cortex (Carter and Ffytche, 2015). More importantly, a previous study has shown that electrical stimulation of the primary visual cortex can elicit phosphenes (Winawer and Parvizi, 2016). Our results provide further evidence of the causal role of the occipital cortex in different types of visual hallucinations.

In this study, the prefrontal cortex yielded no internal or external visual sensations in response to electrical stimulation. One possibility is that the frontal cortex is not involved in the production of visual sensations. It cannot be ruled out that it is difficult to directly elicit experiences from electrical stimulation of frontal sites (Selimbeyoglu and Parvizi, 2010).

### Theoretical Models of Internal and External Visual Sensations

Internal visual sensations are believed to be based on combinations of information retrieved from stored memory (Pearson, 2019). To create internal visual sensations, the initial imagery generation “begins” high in the cortical processing hierarchy, first triggering a cascade of neural events in the prefrontal cortex, then proceeding “backward” to retrieve stored information from the hippocampus, finally forming sensory and spatial representations of the imagery content that can emerge as early as the primary visual cortex. In contrast, the perception attentional dysfunction (PAD) model (Collerton et al., 2005) and misattribution of the internal imagery model (Allen et al., 2008) argue that unbalanced generative perceptions from a widespread network cause external visual sensations.

As mentioned above, internal visual sensations are conceptualized as top-down triggered instances of sensory-memory recall. It naturally follows that higher-level visual areas that are physically closer to the trigger source, such as the hippocampus, would also have stronger perception-like representations, such as external visual sensations, than that of more distant areas, such as V1. We found that internal and external visual sensations had increasing spatial overlap at higher levels along the hierarchical visual cortical stream.

One possibility is that the internal and external visual sensations share common neural mechanisms, as it is more efficient to use the same neural substrates for similar processes. If the occipital visual cortex is hyperactivated, it might be misinterpreted due to faulty interactions with frontoparietal attention networks, whereby images may be perceived in the absence of external stimuli, that is a reduced state of consciousness. Stimulation of the occipital cortex may induce an increase in low-frequency power and/or a decrease in high-frequency power, which is believed to be a reliable neural correlate of unconsciousness (Siclari et al., 2017; Vesuna et al., 2020). Conversely, if the hippocampus and temporal visual cortex in the network are stimulated, one may perceive visual images in the mind (rather than being anchored to the external environment). Future studies will be important to further investigate whether and how specific rhythms contribute to these two distinct processes.

## Limitations

There are several limitations in the present study that need to be considered. First, as mentioned above, the placement of all the electrodes and electrical stimulations is strictly determined according to clinical criteria. Hence, our retrospective analysis could not guarantee a complete sampling of the brain. Second, some areas with greater current thresholds, such as the posterior inferior temporal gyrus or posterior cingulate, elicit visual phenomena that might be due to either local or current propagation to the occipital cortices. Third, the fact that current thresholds that elicited external visual sensations were significantly greater than those of internal visual sensations, reminded us to be cautious about the findings in this study. Finally, there were some potential confounding factors such as complexity and vividness, which may differ between visual hallucination and involuntary imagery. Although there was no significant complexity effect on external visual sensations in the present study, a structured interview after reporting internal and external visual sensations, including additional questions related to the contents, would be helpful in future studies.

## Data Availability Statement

The raw data supporting the conclusions of this article will be made available from the corresponding authors upon reasonable request, without undue reservation.

## Ethics Statement

The present study was reviewed and approved by the Ethical Committee of the Institute of Psychology, Chinese Academy of Sciences (ID H20034). Written informed consent to participate in this study was provided by the participants’ legal guardian/next of kin.

## Author Contributions

YL, LW, and MW conceived of the study. YL and LW designed this study. JW and MW collected data. ZT, YL, and LW analyzed the data. YL and ZT prepared the initial draft of the manuscript. All the authors participated in writing the final draft of the manuscript.

## Conflict of Interest

The authors declare that the research was conducted in the absence of any commercial or financial relationships that could be construed as a potential conflict of interest.

## Publisher’s Note

All claims expressed in this article are solely those of the authors and do not necessarily represent those of their affiliated organizations, or those of the publisher, the editors and the reviewers. Any product that may be evaluated in this article, or claim that may be made by its manufacturer, is not guaranteed or endorsed by the publisher.

## Footnotes

1
http://surfer.nmr.mgh.harvard.edu/
